# Epidermal Cells Expressing Putative Cell Markers in Nonglabrous Skin Existing in Direct Proximity with the Distal End of the Arrector Pili Muscle

**DOI:** 10.1155/2016/1286315

**Published:** 2016-06-08

**Authors:** N. Torkamani, N. W. Rufaut, L. Jones, R. Sinclair

**Affiliations:** ^1^The University of Melbourne, School of Medicine and Dentistry, Department of Dermatology, Melbourne, VIC 3000, Australia; ^2^Epworth Hospital, Dermatology Research Laboratory, Melbourne, VIC 3000, Australia

## Abstract

Inconsistent with the view that epidermal stem cells reside randomly spread along the basal layer of the epidermal rete ridges, we found that epidermal cells expressing stem cell markers in nonglabrous skin exist in direct connection with the distal end of the arrector pili muscle. The epidermal cells that express stem cell markers consist of a subpopulation of basal keratinocytes located in a niche at the lowermost portion of the rete ridges at the distal arrector pili muscle attachment site. Keratinocytes in the epidermal stem cell niche express K15, MCSP, and *α*6 integrin. *α*5 integrin marks the distal end of the APM colocalized with basal keratinocytes expressing stem cell markers located in a well-protected and nourished environment at the lowermost point of the epidermis; these cells are hypothesized to participate directly in epidermal renewal and homeostasis and also indirectly in wound healing through communication with the hair follicle bulge epithelial stem cell population through the APM. Our findings, plus a reevaluation of the literature, support the hierarchical model of interfollicular epidermal stem cell units of Fitzpatrick. This new view provides insights into epidermal control and the possible involvement of epidermal stem cells in nonmelanoma skin carcinogenesis.

## 1. Introduction

In adults, the skin consists of pilosebaceous units (hair follicle and sebaceous glands) and their surrounding epithelium which is referred to as the interfollicular epidermis (IFE). Different groups of stem cells, including epidermal and follicular, are responsible for continuous renewal of skin and its appendages during normal homeostasis and wound repair [1]. One of the major features of stem cells is that they are slowly cycling under normal conditions [2, 3]. While hair growth happens in cycles, sebaceous gland and the epidermal cells replenish continuously. In the epidermis, the basal layer is the only layer which has mitotic activity [4].

Stem cells in the epidermis have been studied widely in mice [1, 5–11] but none of these studies have precisely defined the number of stem cells in the epidermis or their exact location. A stem cell niche has been postulated in the IFE but has never been clearly identified [12]. The location of stem cells in the hair follicle is better characterized; the follicular bulge is a well-known stem cell niche [13, 14]. Bulge cells have an undifferentiated structure [13], have greater in vitro growth ability compared with other cells in the epidermis and follicle [15], and are multipotent [16–20]. They proliferate transiently to initiate hair growth in the early stages of anagen or after being exposed to external simulation [2, 21–23].

The bulge is also the attachment site for the proximal end of the arrector pili muscle (APM), which maintains a close interaction with the bulge throughout the hair growth cycle [24]. The APM has been demonstrated to play an important role in hair follicle integrity and may help to determine the stem cell niche. Moreover, the APM may also function in the bulge to maintain and stimulate these stem cells through Merkel cells [25]. We have recently demonstrated that the APM degenerates in irreversible hair loss conditions such as advanced androgenic alopecia (AGA), but not in reversible alopecia areata (AA) and telogen effluvium (TE) [26, 27], suggesting that the APM is required for maintenance of a functioning stem cell niche in the follicle.

In this study, we hypothesized that there is a similar relationship between the location of stem cells in the IFE and the distal ends of the APM. We have recently demonstrated that the APM can be efficiently stained with phalloidin [28] and attaches to the dermal-epidermal junction at multiple points after dividing into multiple branches. We now report that several stem cell markers colocalize with the distal tips of the APM. The markers evaluated include cytokeratin 15 (K15), *α*6 integrin, and melanoma-associated chondroitin sulfate (MCSP). These results suggest that stem cells in the IFE are located at distinct sites specified by the APM.

Two discrete theories, the hierarchical and stochastic models, have been proposed to explain the nature and organization of epidermal stem cells [10]. The hierarchical model proposes that a discrete population of slow cycling stem cells in IFE undergoes occasional asymmetric division to form one (self-renewing) stem cell and one short-lived transit-amplifying cell (TAC). TACs then undergo multiple rapid and symmetric divisions before differentiating to give rise to postmitotic epidermal cells [1]. On the other hand, the stochastic model suggests that all basal cells are similar and possess random fate. These cells stochastically make a choice between asymmetric and symmetric division, and homeostasis is governed by the probability of each option [29, 30]. Our results support the hierarchical rather than the stochastic model of stem cell organization, because they suggest that IFE stem cells comprise a distinct population defined, at least in part, by their location adjacent to the APM.

## 2. Materials and Methods

### 2.1. Sample Preparation

Skin specimens were obtained from six adult mice (treated according to the guidelines of our institute which conform to the principles of laboratory animal care). Dorsal skin was cut into 5 mm × 5 mm squares, snap-frozen in liquid nitrogen, and stored at −80°C until use.

### 2.2. Immunohistochemistry

OCT embedded samples were cut into 7 *μ*m thick cryosections and then processed for immunohistochemistry. All steps were carried out at room temperature, except for incubation with primary antibodies, which was carried out at 4°C. The sections were air dried and fixed in 3.7% formaldehyde in phosphate buffered saline (PBS) for 10 minutes. Tissue was washed in PBS, 3 times for 5 minutes, and then permeabilized in PBS/0.1% Triton X-100 for 5 minutes. After three PBS washes for 5 minutes the sections were then blocked in 0.3 M glycine/donkey serum (Jackson ImmunoResearch Laboratories, West Grove, PA, USA)/PBS for 60 minutes to minimize nonspecific antibody binding. Following another round of three PBS washes, the application of primary antibody overnight at 4°C was performed.

Triple staining was done on all sections. Primary antibodies were diluted as follows, using PBST/1% BSA (PBS, 0.2% tween-20, 1% bovine serum albumin). Goat anti-human polyclonal antibody against MCSP (aa81-93 Antibody, LSB1596, LSBio, Seattle, WA) was diluted to 1 : 400. The rabbit polyclonal antibodies against *α*6 and *α*5 integrin (Bioss Antibodies, Woburn, MA) were diluted to 1 : 50. The guinea pig anti-K15 antibody (Acris Antibodies, San Diego, CA) was diluted to 1 : 100. The rabbit polyclonal antibody against collagen VII (Bioss antibodies, Woburn, MA) was diluted to 1 : 100.

Sections were then washed 3 times with PBS and subsequently incubated with a mixture of secondary antibodies depending on the applied primary antibodies. Alexa Fluor 594 donkey anti-mouse IgG secondary antibody, Alexa Fluor 488 donkey anti-rabbit IgG secondary antibody, Alexa Fluor 594 Donkey Anti-Goat IgG secondary antibody, Alexa Fluor 594 Goat Anti-Guinea Pig IgG secondary antibody, and Alexa Fluor 488 donkey anti-mouse IgG secondary antibody, all diluted 1 : 2000 using PBST/1% BSA, were applied for 20 minutes. Sections were then costained for 20 minutes with 10 unit/mL DyLight 350 phalloidin (Thermo Scientific, Rockford, IL), diluted in PBS. All stained sections were photographed using an Olympus AX70 Provis microscope and a SPOT Flex 64 MP Color FireWire Digital Camera (Diagnostic Instruments Inc.).

### 2.3. Data Quantification and Statistical Analysis

The epidermis was divided into 50 *μ*m segments in each image. Each segment was scored as positive or negative for expression of phalloidin, K15, MCSP, and *α*5 and *α*6 integrin. The distal strands of the APM have an undulating structure and so can move in and out of the plane of any one section. The extreme tips of all strands cannot be seen in a single section. Hence we considered a segment positive for a phalloidin-stained APM tip when the distal-most visible part of the APM was within 100 *μ*m of the dermoepidermal junction.

The statistical significance of associations between markers was evaluated using Chi-square tests, using Excel 2010 software (Microsoft, Redmond, WA, USA). *P* < 0.05 was considered as significant.

## 3. Results

Expression of the stem cell markers K15, *α*6 integrin, and MCSP was evaluated in the dorsal skin of mice. The APM was stained with a fluorescently labeled phalloidin. The dermoepidermal junction was marked by immunofluorescent staining for collagen VII, a well-known basement membrane marker [31]. *α*5 integrin was evaluated as a marker for the distal ends of the APM [32]. Coexpression of markers was quantified by dividing the epidermis into 50 *μ*m sections and scoring each marker as present or absent in each section. Markers were taken to be coexpressed when they appeared in the same segment.

### 3.1. *α*5 Integrin and Phalloidin Staining Illuminate the Distal Tips of the APM

**Figure 1:**
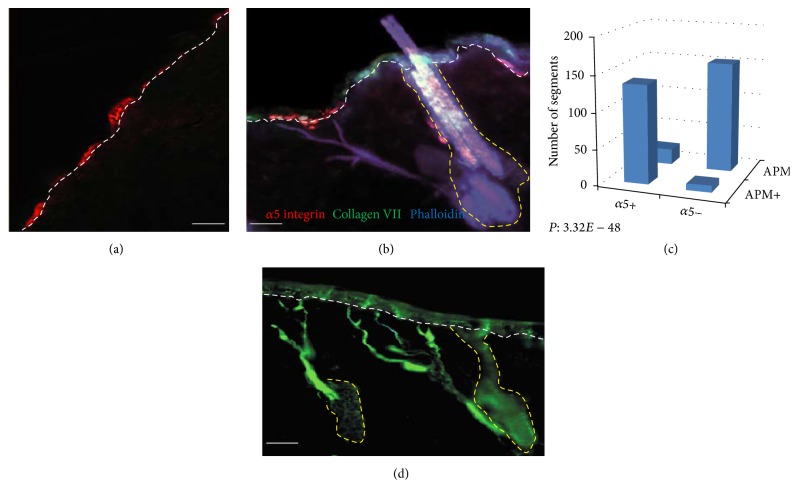
*α*5 integrin and phalloidin staining delineate the distal tips of the arrector pili muscle. Immunofluorescence microscopy showing patterns of phalloidin, expression of *α*5 integrin, and collagen VII. (a-b) *α*5 integrin (red) demonstrates a heterogeneous expression in the basal epidermis. (b) Triple immunofluorescence staining for *α*5 integrin (red), collagen VII (green), and phalloidin (blue) showing marked overlap of *α*5 integrin and phalloidin in the basal epidermis at the distal tip of the arrector pili muscle. (c) Strong correlation between *α*5 integrin expressing cells and cells stained with phalloidin. (d) Extensive branching of the APM (phalloidin-stained) as it ascends and attaches to the dermoepidermal junction. Scale bar: 100 *μ*m.

Patchy distribution of *α*5 integrin positive cells was evident in the IFE (Figure 1(a)). Triple staining for *α*5 integrin, collagen VII, and phalloidin showed that the *α*5 integrin positive cells were located in the basal layer of the epidermis and were consistently located close to the distal ends of the APM (Figure 1(b)). 86.2% of *α*5-positive cells were found in the vicinity of a phalloidin-stained APM tip and 94.5% of phalloidin-stained tips had a nearby *α*5-positive cell (Table 1). The association between *α*5 and phalloidin staining was highly significant (*P* = 3.32 × 10^−48^) (Figure 1(c), Table 1). The APM branched extensively throughout its route towards the epidermis, forming fine strands of muscle at the distal ends (Figure 1(d)). Coexpression of *α*5 integrin, collagen VII, and phalloidin also confirmed our previous finding that the distal APM attaches to the dermal-epidermal junction (Figure 1(b)). In the follicle, *α*5 integrin was expressed in the upper ORS, prominently in the bulge and extending to the upper isthmus (Figure 1(b)).

### 3.2. The Stem Cell Markers, K15, *α*6 Integrin, and MCSP, Localize at the Distal Tips of the APM

**Figure 2:**
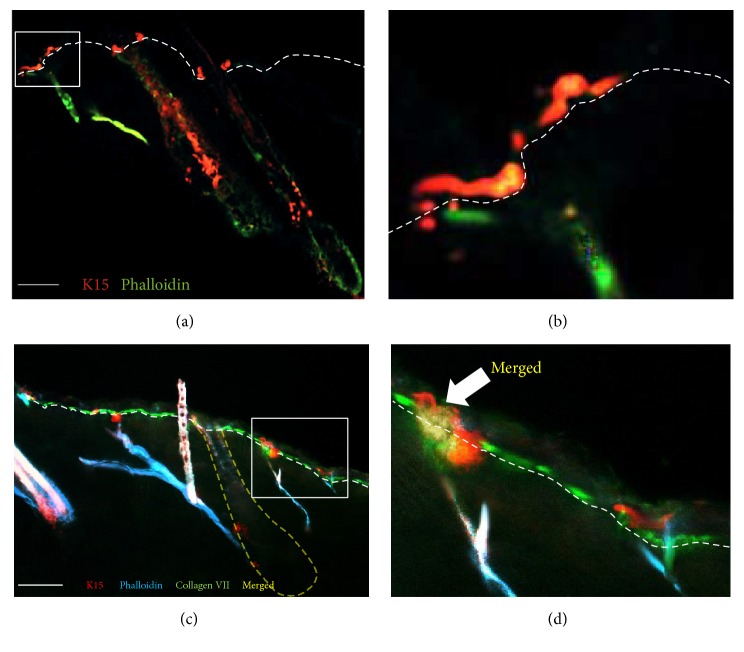
Localization of K15 in the interfollicular epidermis and follicle. Immunofluorescence microscopy showing patterns of phalloidin, expression of *α*5 integrin, K15, and collagen VII. (a) K15 (red) demonstrates a heterogeneous expression in the basal epidermis and follicular bulge. K15 expression is evident at the distal attachment of the APM (green) in the epidermis. (b) Magnified view of the distal attachment of the APM. K15 (red) expression at the distal APM (green) attachment. (c) Triple immunofluorescence staining for K15 (red), collagen VII (green), and phalloidin (blue) showing K15 expressing cells and cells stained with phalloidin in the basal epidermis at the distal tip of the arrector pili muscle (blue) 100 *μ*m.

**Figure 3:**
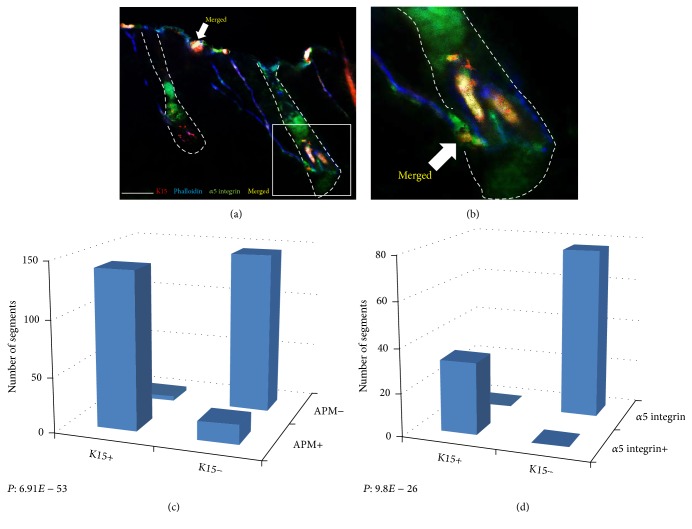
(a) Triple immunofluorescence staining for K15 (red), *α*5 integrin (green), and phalloidin (blue) showing K15 and phalloidin expressing cells in the basal epidermis at the distal tip of the arrector pili muscle (blue). Marked overlap (yellow) of K15 (red) and *α*5 integrin (green) expressing cells in the basal epidermis. (b) Magnified view of the proximal attachment of the APM to the follicle. Expression of K15 and *α*5 integrin at the bulge is evident. (c-d) Strong correlation between K15 and phalloidin and also K15 and *α*5 integrin expressing cells in the epidermis. Scale bar: 100 *μ*m.

K15-positive cells also showed a patchy distribution in the basal layer of the epidermis and were found in close proximity to the distal ends of the APM (Figures 2(a) and 2(b)). Coexpression of K15, phalloidin, and collagen VII confirmed the attachment of the APM to the K15-positive cells in the basal layer of the epidermis (Figures 2(c) and 2(d)). 97.9% of K15 cells were located in close proximity to phalloidin-stained APM tip and 89.3% of phalloidin-stained tips were neighboring K15-positive cells (Table 1). This association was highly significant (*P* = 6.91 × 10^−53^) (Figure 3(c), Table 1). All K15-positive cells were also positive for *α*5 integrin and a strong correlation between the expression of these markers was evident (*P* = 9.8 × 10^−26^) (Figures 3(a), 3(b), and 3(d), Table 1). In hair follicles, K15 was also expressed in the outer root sheath (ORS) at the insertion level of the APM, in the suprabulbar area, and at the follicle opening (Figures 2(a) and 2(b)). K15 expression was stronger in the bulge than in other cells.

**Figure 4:**
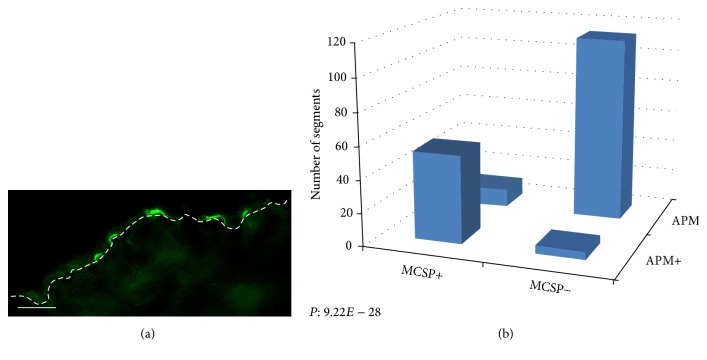
Localization of MCSP in the interfollicular epidermis. Immunofluorescence microscopy showing patterns of expression of MCSP. (a) MCSP (green) demonstrates a heterogeneous expression in the basal epidermis. (b) A strong correlation between phalloidin and MCSP was seen suggesting that cells expressing MCSP in the basal epidermis are located in close proximity to the attachments sites of the APM. Scale bar: 100 *μ*m.

**Figure 5:**
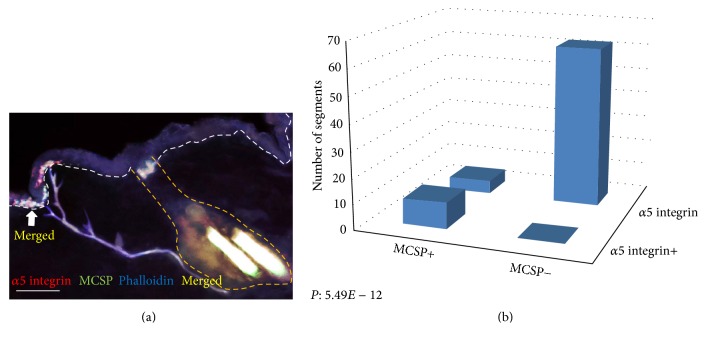
Localization of MCSP in the interfollicular epidermis. Immunofluorescence microscopy showing patterns of expression of MCSP, phalloidin, and K15. (a) Significant MCSP (green) and *α*5 integrin (red) colocalization (yellow) was noted in the interfollicular epidermis. (b) A strong correlation between *α*5 integrin and MCSP was seen suggesting that cells expressing MCSP in the basal epidermis are located in close proximity to the attachments sites of the APM. Scale bar: 100 *μ*m.

MCSP-positive cells were found to be expressed in the epidermis heterogeneously (Figure 4(a)). 83.1% of MCSP-positive cells were found to be in segments which were phalloidin positive and 91.5% of phalloidin-stained tips had a nearby MCSP-positive cell (Table 1). The association between MCSP and phalloidin staining was highly significant (*P* = 9.22 × 10^−28^) (Figure 4(b), Table 1). A restricted epidermal coexpression of *α*5 integrin and MCSP was seen in the arrector pili muscle distal ends (Figures 5(a) and 5(b)). The association between *α*5 integrin and MCSP expression was also significant (*P* = 5.49 × 10^−12^) (Table 1).

**Figure 6:**
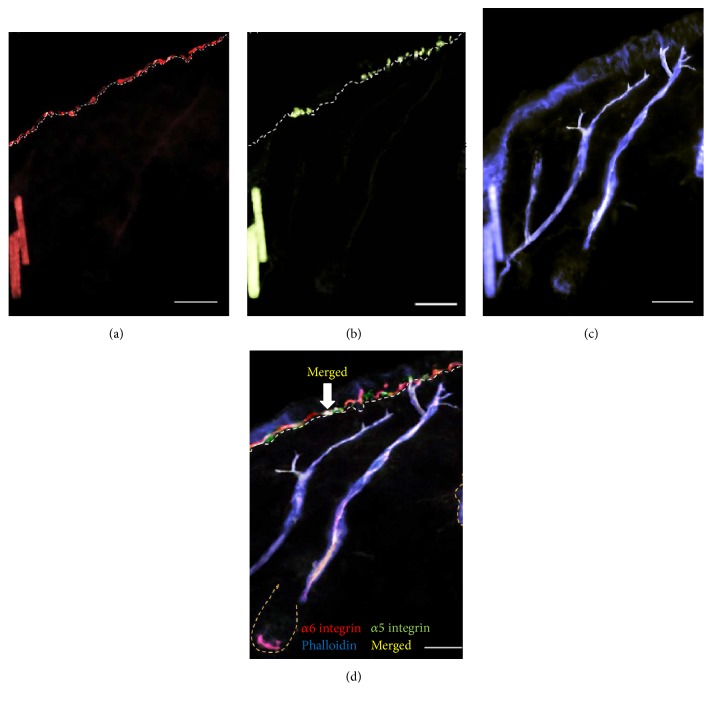
Localization of *α*6 integrin in the interfollicular epidermis and follicle. Immunofluorescence microscopy showing patterns of expression of *α*5 integrin, *α*6 integrin, and phalloidin. (a) *α*6 integrin (red) demonstrates a homogeneous expression in the basal epidermis. (b) Triple immunofluorescence staining for *α*6 integrin (red), *α*5 integrin (green), and phalloidin (blue) showing overlap of *α*6 integrin and *α*5 integrin expressing cells at the distal tips of the APM. (c-d) Due to the homogenous expression pattern of *α*6 integrin it was deemed not to be a suitable marker for IFE stem cells. Scale bar: 100 *μ*m.

*α*6 integrin was detected in a homogenous pattern in the epidermal keratinocytes (Figures 6(a) and 6(b)). These findings make this protein less specific for localization and evaluation of stem cell populations. Multistaining experiments using additional stem cell markers was carried out next.

### 3.3. The Stem Cell Markers Colocalize with Each Other

**Figure 7:**
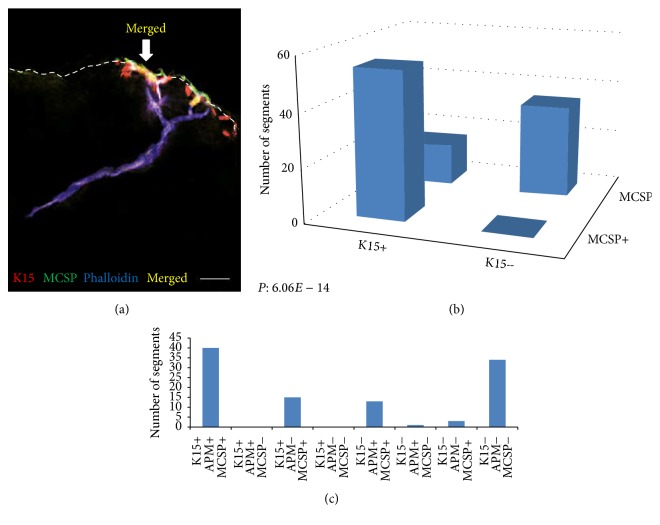
Colocalization of stem cell markers. (a) Marked overlap (yellow) of K15 (red) and MCSP (green) expression in the basal epidermis. (b) A strong correlation between MCSP and K15 was demonstrated. (c) A significant positive three-way correlation was demonstrated between phalloidin (APM), MCSP, and K15 expressing cells. Scale bar: 100 *μ*m.

The expression patterns of the three stem cell markers were associated with each other as well as with the APM tips. All MCSP-positive cells were located in close proximity to cells expressing K15 and 77.5% of K15-positive cells had an adjacent MCSP-positive cell (Figures 7(a) and 7(b), Table 1). The association between cells expressing these markers was significant (*P* = 6.06 × 10^−14^) (Table 1). Costaining with phalloidin demonstrated that 72.7% of K15/MCSP double-positive segments were located near the distal attachment sites of the APM (Figure 7(c), Table 2). The three-way association between K15, MCSP, and phalloidin staining was significant (*P* = 9.70 × 10^−33^) (Table 2). Three-way association between *α*6 integrin and other markers was not done as this marker was considered to be nonspecific due to its wide spread expression in the epidermis.

## 4. Discussion

Although previous studies have investigated the expression of various stem cell markers in the IFE, none have explored their distribution patterns in relation to the APM. Considering that the proximal APM connects to the hair follicle at a well-known stem cell niche, the bulge, we investigated the possible relationship between the distal attachment of the APM and epidermal stem cells. We found that three epidermal stem cell markers colocalized with each other at the distal attachment of the APM to the epidermis [33–38].

Of various putative cell markers that have been used to investigate stem cells in the skin [34, 38–42], we evaluated expression of K15, *α*6 integrin, and MCSP. K15 is a type I keratin expressed in the follicle bulge [43, 44], keratinocytes of the outer root sheath located above the hair bulb, and basal keratinocytes of the epidermis [36]. Bulge K15-positive cells are label retaining and slow cycling and are able to regenerate all follicular lineages. Hence, K15 has become one of the most widely used markers for stem cells in the bulge [45]. K15 is also expressed intermittently along the basal layer of the epidermis [36].

Cells expressing *α*6 integrin display prominent stem cell characteristics [34]. *α*6 integrin plays a significant role in maintaining epithelial integrity, as its absence results in a broad range of epithelial defects such as epidermolysis bullosa [46]. While *α*6 integrin has been demonstrated to be expressed by stem cells, transient amplifying is also known to express high levels of *α*6 integrin hence it has been suggested that this marker cannot be used as a solo epidermal stem cell probe in mice. The results of this study confirmed this and multimarker staining techniques with two other stem cell markers, K15 and MCSP, were implemented to overcome this limitation of *α*6 integrin.

Melanoma-associated chondroitin sulfate (MCSP) has been demonstrated to be associated with stem cells in the epidermis [38]. NG2, the murine homolog of MCSP [12], is expressed in undifferentiated cells with mitotic potential including the follicular bulge and ORS [47]. As these cells differentiate, NG2 expression is downregulated [48]. Furthermore, MCSP-positive cells have been shown to colocalize with *β*1 integrin bright cells which are well-known stem cells [38]. Considering these characteristics, MCSP has been suggested to be a good epidermal stem cell marker [38].

Colocalization of these markers supports the identity of labeled cells as stem cells. Waseem et al. demonstrated that cells expressing K15 have a strong reactivity with *α*6 integrin positive basal keratinocytes with premitotic activity [36]. MCSP and K15 also showed a patchy coexpression along the basal epidermis, mostly with a punctate pattern [35, 36]. We similarly found a strong correlation between these stem cell markers. K15 and *α*6 integrin positive cells were colocalized. MCSP-positive cells and K15-positive cells in the IFE also expressed *α*6 integrin, although *α*6 integrin is not limited to these clusters and has a more extensive distribution. We quantified these expression patterns and showed that the associations between the three markers were highly significant.

The majority of stained stem cells in the IFE were located at the distal tips of the APM. We used phalloidin and *α*5 integrin staining to identify the distal tips. *α*5*β*1 integrin expression has previously been described in association with the distal APM. It is expressed in the epidermal basal layer and binds to fibronectin in the extracellular matrix [32, 33]. In humans, we have recently found that the distal APM attachment is mostly located at the base of the rete ridges. It has been demonstrated that stem cells in human epidermis reside in the base of the rete ridges [49]. Thus human epidermal stem cells may also be located at the tips of the APM.

A stem cell niche has been postulated in the IFE but has never been clearly identified. Based on the results from this study it is suggested that a stem cell niche exists in the interfollicular epidermis in close proximity to the distal ends of the epidermis APM. The proximal end of arrector pili muscle has been suggested to play a significant role in maintaining the stem cell niche in the bulge and is also involved in sustaining the bulge cell's undifferentiated state. Moreover the cellular population in the distal and proximal ends of the arrector pili muscle shares some characteristics such as being in close proximity to Merkel cells. Merkel cells have been suggested to be involved in maintenance of stem cell populations as well as being tactile receptors.

Considering the proximity of the IFE stem cell populations identified in this study to the APM and also previous findings regarding the role of the APM in maintenance of the stem cell niche in the bulge and the proximity of both ends of the muscle to Merkel cells it is suggested that the IFE stem niche identified here shares similarities with the previously well-established bulge niche. Keratinocyte stem cells have been found to respond to mechanical signals that change their shape, raising the possibility that APM-mediated tension regulates stem cells in both the bulge and the IFE. There is growing evidence that stem cells respond to mechanical as well as biochemical signals. Mechanical stimuli such as stretching, matrix stiffness, and compressive stresses regulate stem cell differentiation and fate through an organized cascade of biochemical signals. Hence APM-mediated forces may contribute to the regulation of stem cells in both the follicle bulge and the IFE. In the context of a wound, disruption of the APM may contribute to the activation of remnant stem cells via altered mechanical signaling. Further investigation of the role of the APM in skin homeostasis and wound healing would be worthwhile.

Hierarchical and stochastic models of stem cell organization in the skin have been proposed [10]. The hierarchical model proposes the existence of discrete stem and transit-amplifying (TA) cell populations, leading to the generation of an epithelial proliferation unit (EPU). The stochastic model proposes random fate for a single population of committed progenitor cells, which have the ability to divide symmetrically or asymmetrically based on environmental conditions. Studies involving lineage tracing of basal cells in mice have been in favor of the stochastic model. However these studies, which targeted the epidermal skin in various sites including the mouse tail, hind paw, and ear, do not specify whether the implemented labeling techniques were random or stem cell clones were specifically marked. As mentioned previously selective labeling of stem cell clones and differentiating them from transient ones is a key step in lineage studies which if not implemented accurately can result in imprecise and biased results. Furthermore, the stochastic model cannot fully explain epidermal regeneration upon insult and the role of the IFE stem cells in wound healing. A more recent study using Cre-lineage tracers in mouse tail supports the hierarchical model. Using two distinct Cre drivers, this study confirmed the existence of a heterogeneous pool of epidermal progenitors with different life span profiles. These cells form a hierarchical stem cell population and contribute to epidermal homeostasis, consolidating the traditional TAC model with the stochastic hypothesis.

Restricted location of stem cells at a distinct physiological location, the tips of the APM, is more consistent with a discrete stem cell population rather than presence of stochastic stem cell behavior among a more widely distributed population. K15 and *α*6 integrin positive cells have previously been suggested to possess stem cell like potential and undergo asymmetric division. The strong colocalization K15/MCSP of certain populations of basal cells supports the existence of at least two cell populations, epidermal progenitors and TACs. Moreover the findings support the existence of EPUs which possess asymmetrical division capacity at the distal attachment of the APM to IFE. The distal APM may also be involved in maintenance of this niche, analogous to the interaction between the proximal APM and the bulge. More specifically, the APM may support, protect, or stimulate stem cell populations in both the bulge and epidermis.

## What Is Already Known about This Topic?

- The follicle bulge, the proximal arrector pili muscle (APM) attachment, is a well-known stem-cell niche.
- A stem cell niche has been postulated in the interfollicular epidermis but has never been clearly identified.

## What Does This Study Add?

- Interfollicular epidermal stem cells are restricted to basal keratinocytes, mostly near the distal attachment site of APM.
- The distal APM may contribute to the formation of a niche for epidermal stem cells, as the proximal muscle does for follicular stem cells.

## Figures and Tables

**Table 1:** Colocalization of APM and stem cell markers.

	MCSP+	MCSP−		K15+	K15−	
Phalloidin+	54	5		142	17	
Phalloidin−	11	113		3	145	
			*P* = 9.2 × 10^−28^			*P* = 6.9 × 10^−53^

	MCSP+	MCSP−		K15+	K15−	

*α*5+	10	0		33	0	
*α*5−	5	62		0	77	
			*P* = 5.5 × 10^−12^			*P* = 9.8 × 10^−26^

	MCSP+	MCSP−				

K15+	55	16				
K15−	0	35				
			*P* = 6.1 × 10^−14^			

**Table 2:** Three-way association of APM and stem cell markers.

		K15+	K15−	
Phalloidin+	MCSP+	40	13	
Phalloidin+	MCSP−	0	1	
Phalloidin−	MCSP+	15	3	
Phalloidin−	MCSP−	0	34	
				*P* = 9.7 × 10^−33^

## Abbreviations

APM: Arrector pili muscle
K15: Cytokeratin 15
MCSP: Melanoma-associated chondroitin sulfate
IFE: Interfollicular epidermis
TAC: Transit-amplifying cell
AGA: Androgenic alopecia
AA: Alopecia areata
TE: Telogen effluvium
ECM: Extracellular matrix
EPU: Epithelial proliferation unit
CP: Committed progenitor
PBS: Phosphate buffered saline.
